# Hypoxia increases neutrophil-driven matrix destruction after exposure to *Mycobacterium tuberculosis*

**DOI:** 10.1038/s41598-018-29659-1

**Published:** 2018-07-31

**Authors:** Catherine W. M. Ong, Katharine Fox, Anna Ettorre, Paul T. Elkington, Jon S. Friedland

**Affiliations:** 1 Infectious Diseases and Immunity, Hammersmith Campus, Imperial College London, London, UK; 2 Division of Infectious Diseases, Department of Medicine, Yong Loo Lin School of Medicine, National University of Singapore, Singapore, Singapore; 3 NIHR Biomedical Research Centre, Faculty of Medicine, University of Southampton, Southampton, UK

## Abstract

The importance of neutrophils in the pathology of tuberculosis (TB) has been recently established. We demonstrated that TB lesions in man are hypoxic, but how neutrophils in hypoxia influence lung tissue damage is unknown. We investigated the effect of hypoxia on neutrophil-derived enzymes and tissue destruction in TB. Human neutrophils were stimulated with *M*. *tuberculosis (M*.*tb)* or conditioned media from *M*.*tb*-infected monocytes (CoMTB). Neutrophil matrix metalloproteinase-8/-9 and elastase secretion were analysed by luminex array and gelatin zymography, gene expression by qPCR and cell viability by flow cytometry. Matrix destruction was investigated by confocal microscopy and functional assays and neutrophil extracellular traps (NETs) by fluorescence assay. In hypoxia, neutrophil MMP-8 secretion and gene expression were up-regulated by CoMTB. MMP-9 activity and neutrophil elastase (NE) secretion were also increased in hypoxia. Hypoxia inhibited NET formation and both neutrophil apoptosis and necrosis after direct stimulation by *M*.*tb*. Hypoxia increased TB-dependent neutrophil-mediated matrix destruction of Type I collagen, gelatin and elastin, the main structural proteins of the human lung. Dimethyloxalylglycin (DMOG), which stabilizes hypoxia-inducible factor-1α, increased neutrophil MMP-8 and -9 secretion. Hypoxia in our cellular model of TB up-regulated pathways that increase neutrophil secretion of MMPs that are implicated in matrix destruction.

## Introduction

The clinical features of tuberculosis (TB) are increasingly recognised to be mediated by the host innate immune response^1–3^. Proteases such as matrix metalloproteinases (MMPs) and neutrophil elastase have been implicated in TB immune-mediated pathology^1,4,5^. Neutrophil derived MMP-8 is increased in TB patients and is associated with clinical disease severity^6^, while neutrophil elastase may have anti-mycobacterial activities^7^. Targeting host proteases is an attractive therapeutic option to minimize the consequence of *M*. *tuberculosis* (*M*.*tb*) infection, which commonly results in cavity formation, bronchiectasis and scarring leading to patient morbidity even with appropriate antibiotic treatment^8^.

Hypoxia affects diverse aspects of the host immune response in TB. Tuberculous granulomas are hypoxic both in murine models and in humans, as we demonstrated in TB patients using (18 ^F^)-MISO PET/CT scanning^4,9^. In TB, hypoxia initiates the angiogenesis required for the formation of granulomas^10^. Inhibition of vascular endothelial growth factor limits infection burden and dissemination, and improves oxygenation to granulomas by normalizing vasculature in *M*. *marinum*-infected zebrafish^10,11^. The effect of hypoxia on the pathogen is diverse. Human β-defensin-2 is upregulated in hypoxia in macrophages, inhibiting intracellular mycobacterial growth^12^. Conversely, hypoxia increases survival fitness in *M*.*tb* with the microbe adapting to hypoxia^13^, inducing a non-replicating state or dormancy to become less susceptible to anti-mycobacterial treatment^14^.

Neutrophils, which secrete MMP-8,-9 and neutrophil elastase, are abundant in the respiratory secretions of TB patients^15^. Neutrophil recruitment is associated with increased pathology in TB animal models, and the unique TB gene signature detected in patients reflects neutrophil activity^6,16–18^. In hypoxia, human neutrophils demonstrate decreased apoptosis and respiratory burst^19^. Hypoxia-inducible factor-α (HIF-α), activated in neutrophils in states of hypoxia and infection, prolongs the life-span of leukocytes, reduces bacterial burden and enhances reactive nitrogen species in neutrophils prior to infection in a mycobacterial model in zebrafish^20,21^. Furthermore, hypoxia decreases apoptosis and increases life-span in human neutrophils^22^.

The relationship between hypoxia and neutrophil-driven pathology in TB has not been investigated and mechanisms by which neutrophils may increase inflammatory tissue damage are poorly defined. We hypothesize that a hypoxic environment exacerbates neutrophil-dependent immunopathology in TB. We show that hypoxia increases neutrophil MMP-8, -9 and neutrophil elastase secretion in TB, which then drive matrix destruction. Increased protease activity is dependent on the HIF-1α pathway. Conversely, the production of NETs is suppressed by hypoxia. Taken together, the data show that hypoxic neutrophils increase immune-mediated pathology by up-regulating enzymes that destroy the extracellular matrix in TB by a HIF-1α-dependent mechanism.

## Materials and Methods

### *M*.*tb* culture

*M*. *tuberculosis* H37Rv was cultured in supplemented Middlebrook 7H9 medium (BD Biosciences). For infection experiments, mycobacteria were used at mid-logarithmic growth at an optical density of 0.60 (Biowave cell density meter; WPA). All *M*.*tb* related experiments were performed in a Containment Level 3 facility.

### Cell culture and stimulation

Ethical approval for obtaining healthy human volunteer blood to extract human neutrophils was provided by the Outer West London Research Ethics Committee (REC reference 09/H0709/46). All research was performed in accordance with relevant guidelines/regulations, and informed consent was obtained from all participants. Whole blood were drawn in preservative-free heparin and mixed with equal volumes of 3% dextran saline to remove erythrocytes. Neutrophils were isolated from the resulting cell suspension using Ficoll-Paque density centrifugation and three rounds of hypotonic lysis. Neutrophil purity was over 95% by FACS and viability >99% by trypan blue assay. In some experiments, neutrophils were pre-incubated with 1 mM dimethyloxalylglycine, DMOG (Sigma) or HIF-1α inhibitor KC7F2 (Tocris Bioscience) for 30 minutes before stimulation. In all experiments involving live *M*. *tuberculosis* H37Rv, tissue culture medium was sterile filtered through 0.2-μm Durapore membranes (Millipore) before removing from the CL3 laboratory, which does not remove MMPs^23^. Neutrophils were stimulated at 37 °C in normoxia (21% oxygen) or hypoxia (1% oxygen) in a hypoxic incubator (Galaxy 14 S, New Brunswick, Eppendorf).

Primary human blood monocytes were prepared from donor leukocyte cones of residual cells from blood donation of healthy donors (National Blood Transfusion Service, UK). After density gradient centrifugation (Ficoll Paque) followed by adhesion purification, monocyte purity was over 95% by FACS analysis. Monocytes were infected with *M*.*tuberculosis* at a multiplicity of infection (MOI) of 1. After incubation at 37 °C for 24 hours, conditioned medium was harvested and was termed CoMTB. Media from uninfected monocytes was termed CoMCont.

### Neutrophil elastase ELISA

Neutrophil elastase concentration was measured with the Human Elastase ELISA kit (Hycult biotech, Uden, The Netherlands), with a minimum level of detection of 0.4 ng/ml. The manufacturer’s protocol was followed and the plate read at 450 nm using a microplate reader (µQuant, Biotek Instruments, UK).

### Luminex array

MMP-8 and -9 concentrations were analyzed by Fluorokine multianalyte profiling kit according to the manufacturer’s protocol (R&D Systems) on the Luminex 200 platform (Bio-Rad). The minimum level of detection for MMP-8 and -9 was 110 pg/ml and 65 pg/ml respectively.

### Matrix degradation assay

Type I collagen and elastin degradation was assessed using the EnzChek ® Gelatinase/Collagenase Assay kit and the EnzChek ® Elastase Assay kit (Molecular Probes) respectively. Samples were activated with 2 mM of 4-amino-phenyl mercuric acetate (APMA) for 1 hour at 37 °C. 80 µL of reaction buffer were added with 20 µL of DQ collagen or elastin (Invitrogen) to a final concentration of 25 µg/ml and 100 μg/ml respectively. Activated samples were subsequently added, and activity detected at specified times using a fluorometer (FLUOstar Galaxy).

### Gelatin zymography

This was performed as previously described^24^. Densitometric image analysis was performed using Scion Image version Beta.4.0.2.

### Isolation and quantification of neutrophil extracellular traps (NETs)

Human neutrophils were infected with *M*.*tb* at an MOI of 10, while 20 nM PMA was used as a positive control. 5 U/ml of micrococcal nuclease (Fermentas) was added in each well for 10 minutes at 37 °C, after which EDTA was used to halt the reaction. Supernatants were collected, sterile filtered and stored at 4 °C. NETs were quantified using QuantiT PicoGreen (Invitrogen) according to manufacturer’s instructions.

### Real-time PCR

Total RNA was extracted from 2 × 10^6^ neutrophils using the RNeasy Mini Kit (Qiagen). Quantitative real-time RT-PCR was performed using the OneStep RT- PCR master mix (Qiagen) according to the manufacturer’s instruction on a Stratagene Mx3000P platform using 5–10 µg per sample. MMP-8, neutrophil elastase and β-actin primer and probe mixes were obtained from Applied Biosystems. MMP-9 forward and reverse primers were 5′-AGGCGCTCATGTACCCTATGTAC and 5′- GCCGTGGCTCAGGTTCA respectively, and the probe was 5′-FAM-CATCCGGCACCTCTATGGTCCTCG-TAMRA. To accurately determine the quantitative change in RNA, standard curves were prepared from plasmids subjected to real-time PCR as above. MMP-8 and -9 data were normalized to β-actin detected in the same sample.

### Flow cytometry

Cell viability was assessed by staining neutrophils with Annexin V and propidium iodide using Annexin V-FITC apoptosis detection kit (eBioscience, Affymetrix, California, USA) and live/dead fixable stain kit (Invitrogen). Neutrophils were stimulated with 200 ng/ml staurosporine to induce apoptosis and this was used as a positive control for all experiments. Annexin V was detected on the FL-1 channel with propidium iodide and live/dead fixable dead cell stain kit on FL-3. A total of 50,000 events were gated and analysed on BD FACSCalibur flow cytometer using CellQuest. Data was analysed using FlowJo 7.6.5 (Tree Star).

### Immunofluorescence microscopy

Permanox chamber slides (Nunc Labtech) were coated with 0.1 mg/ml fibrinogen with or without 25 µg/ml of DQ collagen or DQ Elastin for 60 minutes. Neutrophils were stimulated in normoxia or hypoxia, chambers removed and cover slips were placed prior to imaging. For experiments involving *M*.*tb*, neutrophils were infected with an MOI of 10. Samples were fixed with 4% paraformaldehyde for 30 minutes and permeabilized with 0.5% saponin for 10 minutes. Chambers were removed, slides stained with auromine-rhodamine (Merck Chemicals) and Fluoroshield Mounting medium with DAPI (Abcam) were added. Images were captured using Leica confocal microscope (Leica TCS SP5) and processed using Leica LAS AF Lite 2.6.0 (Leica Microsystems, Germany) and ImageJ 1.43U (NIH, USA). Phagocytic index was the ratio of number of neutrophils that contain at least 1 *M*.*tb* bacilli to the total number of neutrophils in 1 field multiplied by 100%.

### Statistical analyses

Data were analyzed using GraphPad Prism (version 5.04, GraphPad Software). Data are expressed as mean ± s.e.m. unless stated otherwise. All experiments are performed in technical triplicates on a minimum of 2 donors studied on separate occasions. All data points of 2 donors each in triplicate are presented. For FACS analysis, a minimum of 4 donors were studied on separate occasions with bars representing mean ± s.e.m. Multiple intervention experiments are compared with one-way ANOVA followed by Tukey’s post-test correction, kinetics experiments were analysed by 2-way ANOVA with Bonferonni post-test while continuous variables between 2 sets of data are assessed using two-tailed Mann-Whitney-U test. *P* values of less than 0.05 are taken as statistically significant.

### Data availability

The datasets generated during and/or analysed during the current study are available from the corresponding author on reasonable request.

## Results

### Hypoxia increases TB-driven neutrophil collagenase (MMP-8) and neutrophil elastase secretion

**Figure 1:**
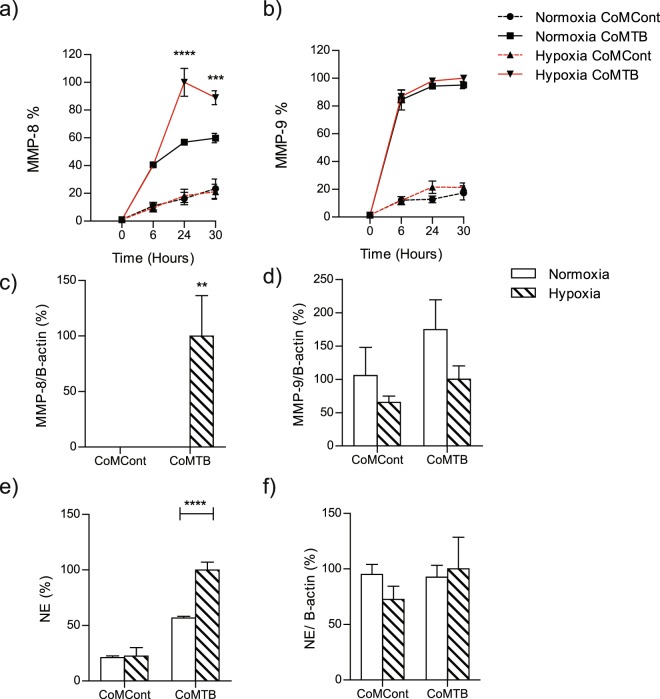
Hypoxia increases MMP-8 and neutrophil elastase secretion from CoMTB-stimulated neutrophils. (**a**) Neutrophil MMP-8 and (**b**) MMP-9 secretion in normoxia and hypoxia with CoMCont (control) and CoMTB stimulation. (**c**) MMP-8 gene expression is increased in hypoxia at 24 h. (**d**) MMP-9 gene expression is unchanged with hypoxia at 24 h. (**e**) Neutrophil elastase (NE) secretion is increased in CoMTB-stimulated neutrophils in hypoxia. (**f**) Neutrophil elastase gene expression is unaffected by oxygen concentration. **p < 0.01, ***p < 0.001, ****p < 0.0001 by 2-way ANOVA with Bonferonni post-test. Bars represent mean ± s.e.m of all data points from 2 donors each in triplicate, normalized to hypoxia CoMTB.

First, we investigated the effects of hypoxia on monocyte-dependent networks that drive neutrophil MMP and elastase secretion in TB. CoMTB upregulated secretion of both neutrophil collagenase MMP-8 and gelatinase MMP-9 secretion in normoxia. Hypoxia increased MMP-8 secretion to 100% at 24 hours which decreased slightly to 89% at 30 hours (*p* < 0.0001 and p < 0.001 respectively) (Fig. 1a). MMP-9 secretion was independent of oxygen tension (Fig. 1b). Similarly, hypoxia increased MMP-8 but not MMP-9 gene expression (Fig. 1c,d respectively). Neutrophils stimulated with CoMTB secreted neutrophil elastase, with hypoxia increasing the secretion by a further 76% (*p* < 0.0001, Fig. 1e). Neutrophil elastase gene expression was unchanged between normoxia and hypoxia (Fig. 1f).

### Hypoxia does not alter viability of CoMTB-stimulated neutrophils

**Figure 2:**
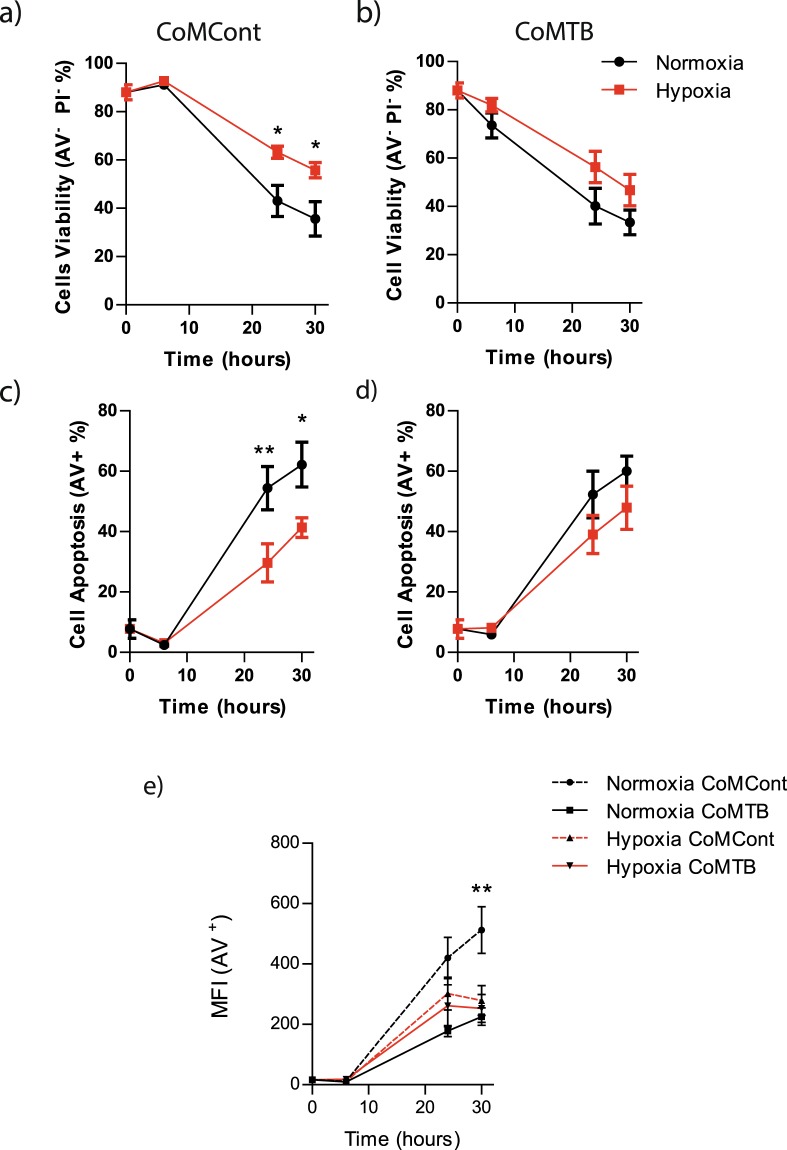
CoMTB abrogates increased neutrophil viability found in hypoxia. (**a**) Neutrophil viability is increased in hypoxia in CoMCont (control) neutrophils. (**b**) Neutrophil viability in the presence of CoMTB is not affected by oxygen tension. (**c**) Neutrophil apoptosis is decreased in hypoxia in CoMCont neutrophils. (**d**) Neutrophil apoptosis after CoMTB stimulation is independent of oxygen tension. (**e**) Annexin V mean fluorescence intensity is increased in CoMCont-stimulated neutrophils in normoxia compared to hypoxia but not in CoMTB-stimulated neutrophils, demonstrating that CoMTB negates the effect of oxygen tension on neutrophil viability. *p < 0.05, **p < 0.01 by 2-way ANOVA with Bonferonni post-test. Bars represent mean ± s.e.m of n = 5 neutrophil donors on 5 separate occasions.

Next, we investigated whether the increase in CoMTB-dependent neutrophil MMP-8 secretion in hypoxia was due to altered cellular viability. Over 30 hours, the viability of control neutrophils stimulated by CoMCont declined from 88% ± 3% (mean ± s.e.m) to 35% ± 7% in normoxia. Viability at 30 hours was higher at 55% ± 3% in the presence of hypoxia in control neutrophils (p < 0.05, Fig. 2a). In CoMTB-stimulated neutrophils, viability similarly declined in normoxia to 33% ± 5%, but although viability increased to 47% ± 7% in hypoxia, the effect of decreased oxygen tension was less pronounced (Figs 2b, S1). The proportion of apoptotic control neutrophils in normoxia at 30 hours was 62% ± 7, with hypoxia decreasing apoptosis to 41% ± 3 (*p* < 0.05, Fig. 2c). CoMTB stimulation negated the effects of hypoxia on cellular apoptosis (Fig. 2d). Mean fluorescence intensity of Annexin V, indicating cellular death, was increased in control neutrophils at 30 hours in normoxia compared to hypoxia (CoMCont normoxia 512 A.U. ± 77 vs CoMCont hypoxia 279 ± 49, p < 0.01), but this effect was absent on stimulation with CoMTB (Fig. 2e). Although studying significantly more donors might generate a statistical significant difference, the effect on viability seems less pronounced in the context of CoMTB stimulation. Together, these data show that increased neutrophil MMP-8 secretion in hypoxia after CoMTB stimulation was not due to increased neutrophil viability.

### Hypoxia decreases formation of neutrophil extracellular traps (NETs) after direct *M*.*tb* infection

**Figure 3:**
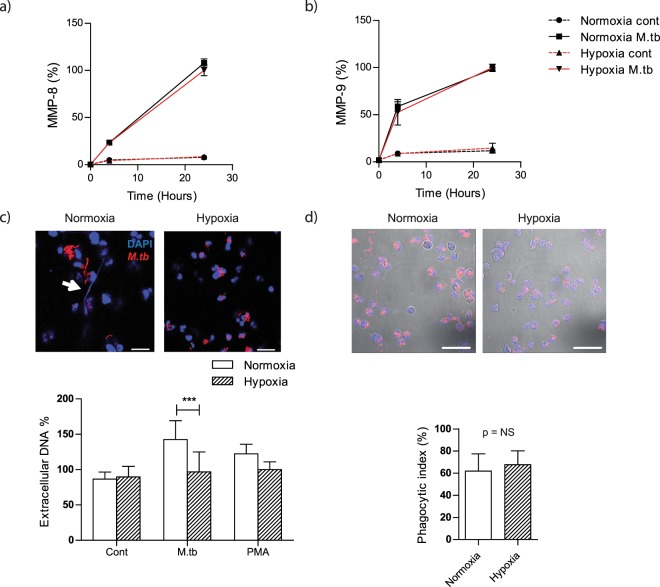
Hypoxia does not affect neutrophil MMP secretion in direct *M*.*tb* infection, suppresses NET formation and does not affect neutrophil phagocytosis. (**a**) MMP-8 and (**b**) MMP-9 secretion is upregulated in *M*.*tb*-infected neutrophils but is independent of oxygen tension. (**c**) Hypoxia decreases production of NETs by *M*.*tb*-infected neutrophils as demonstrated by quantitative fluorescence imaging and confocal microscopy. White arrow on confocal image indicating NETs. Scale bar 20 μm. ***p < 0.001 by 2-way ANOVA with Bonferonni post-test. (**d**) Neutrophil phagocytosis is independent of hypoxia. Neutrophils were infected with *M*.*tb* MOI 10 for 4 hours in normoxia or hypoxia, fixed and stained with Auramine-Rhodamine (red) for *M*.*tb* and DAPI (blue) for neutrophil nuclei. Neutrophils from 10 different fields were counted and phagocytic index analysed by Mann-Whitney test. Bars represent mean ± s.e.m of all data points from 2 donors each in triplicate normalized to hypoxia *M*.*tb* for MMP-8 and -9, and normalized to hypoxia PMA for extracellular DNA.

We then focused on direct *M*.*tb* infection of neutrophils and evaluated the secretion of neutrophil MMPs over time. Neutrophil MMP-8 and -9 secretion increased for 30 hours after *M*.*tb* infection, but this was not altered in the presence of hypoxia (Fig. 3a,b). In contrast, the release of NETs after infection with *M*.*tb*, was decreased by 32% (*p* < 0.001) in hypoxia compared to normoxia at 4 hours, with confocal microscopy showing a decrease in NET formation (Fig. 3c). Neutrophil extracellular traps (NETs) are produced when neutrophils encounter *M*.*tb*^25^, and they may aid in phagocytosis of pathogens^26^. However, when we compared neutrophil phagocytosis of *M*.*tb* in hypoxia versus normoxia at 4 hours, there was no difference in the phagocytic index (Fig. 3d). A later time point was not used as neutrophil viability diverges.

### Hypoxia increases neutrophil viability in *M*.*tb*-stimulated and non-stimulated neutrophils

**Figure 4:**
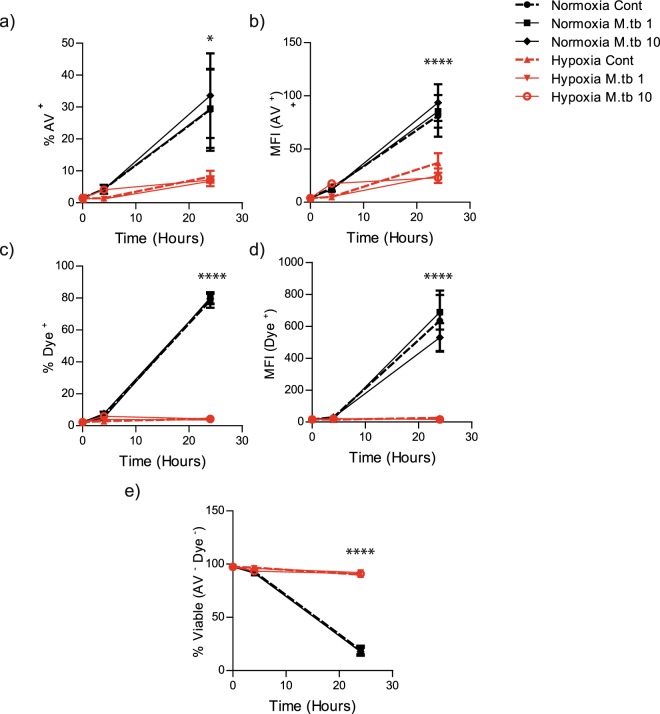
Hypoxia delays apoptosis, necrosis and increases neutrophil viability in *M*.*tb* infection. (**a**) The percentage of apoptotic neutrophils (Annexin V positive) is lower in hypoxia than normoxia for uninfected and infected cells. *p < 0.05 between normoxia *M*.*tb* 10 and hypoxia *M*.*tb* 10 by 2-way ANOVA with Bonferonni post-test. (**b**) Mean fluorescent intensity of Annexin V positive cells was higher in cells in normoxia than in hypoxia. ****p < 0.001 between normoxia control vs. hypoxia control; normoxia *M*.*tb* 1 vs. hypoxia *M*.*tb* 1; and normoxia *M*.*tb* 10 vs. hypoxia *M*.*tb* 10. (**c**) The proportion of necrotic cells stained positive for Live-Dead dye was lower in hypoxia than normoxia for uninfected and infected cells. ****p < 0.001 between normoxia control vs. hypoxia control; normoxia *M*.*tb* 1 vs. hypoxia *M*.*tb* 1; and normoxia *M*.*tb* 10 vs. hypoxia *M*.*tb* 10. (**d**) Mean fluorescent intensity of Live-Dead dye positive cells was higher in infected and uninfected cells in normoxia than in hypoxia. ****p < 0.001 between normoxia control vs. hypoxia control; normoxia *M*.*tb* 1 vs. hypoxia *M*.*tb* 1; and normoxia *M*.*tb* 10 and hypoxia *M*.*tb* 10. (**e**) The proportion of viable cells is higher in hypoxia than normoxia for uninfected and infected cells, analysed by Annexin V staining. ****p < 0.001 between normoxia control vs. hypoxia control; normoxia *M*.*tb* 1 vs hypoxia *M*.*tb* 1; and normoxia *M*.*tb* 10 vs hypoxia *M*.*tb* 10. Bars represent mean ± s.e.m of n = 4 neutrophil donors on 4 separate occasions.

Since the NET effect could have been due to altered cell viability, and apoptosis of unstimulated neutrophils is delayed in hypoxia^19,22^, we also investigated the effects of *M*.*tb* infection on neutrophil apoptosis and cell viability in hypoxia. Analysis of primary human neutrophils at 24 hours showed delayed apoptosis with *M*.*tb* infection in hypoxia (*M*.*tb* in normoxia 29% ± 12 s.e.m. vs hypoxia 7% ± 2, p < 0.05, Fig. 4a) and decreased Annexin V mean fluorescence intensity (*M*.*tb* in normoxia 85 A.U. ± 15 vs hypoxia 25 ± 7, p < 0.001, Fig. 4b). Similarly in hypoxia, there were far fewer necrotic *M*.*tb*-infected neutrophils (*M*.*tb* in normoxia 80% ± 4 vs hypoxia 4% ± 1, p < 0.001, Fig. 4c) and decreased live-dead dye mean fluorescence intensity (*M*.*tb* in normoxia 688 A.U. ± 108 vs hypoxia 17 ± 5, p < 0.001 Fig. 4d). The delay in neutrophil apoptosis and necrosis at 24 hours translated to a 400% increase in neutrophil viability (Annexin V and Dye negative) in hypoxia compared to normoxia (*M*.*tb* in normoxia 18% ± 4 vs hypoxia 92% ± 2 *p* < 0.001, Fig. 4e). Apoptosis, necrosis, cell viability and mean fluorescence intensity of Annexin V and live-dead dye were all independent of *M*.*tb* multiplicity of infection (MOI), (Figs 4 and S2). Thus, the decrease in NETs in hypoxia was not due to a decrease in neutrophil viability.

### Neutrophil-driven matrix destruction in TB is increased with hypoxia

**Figure 5:**
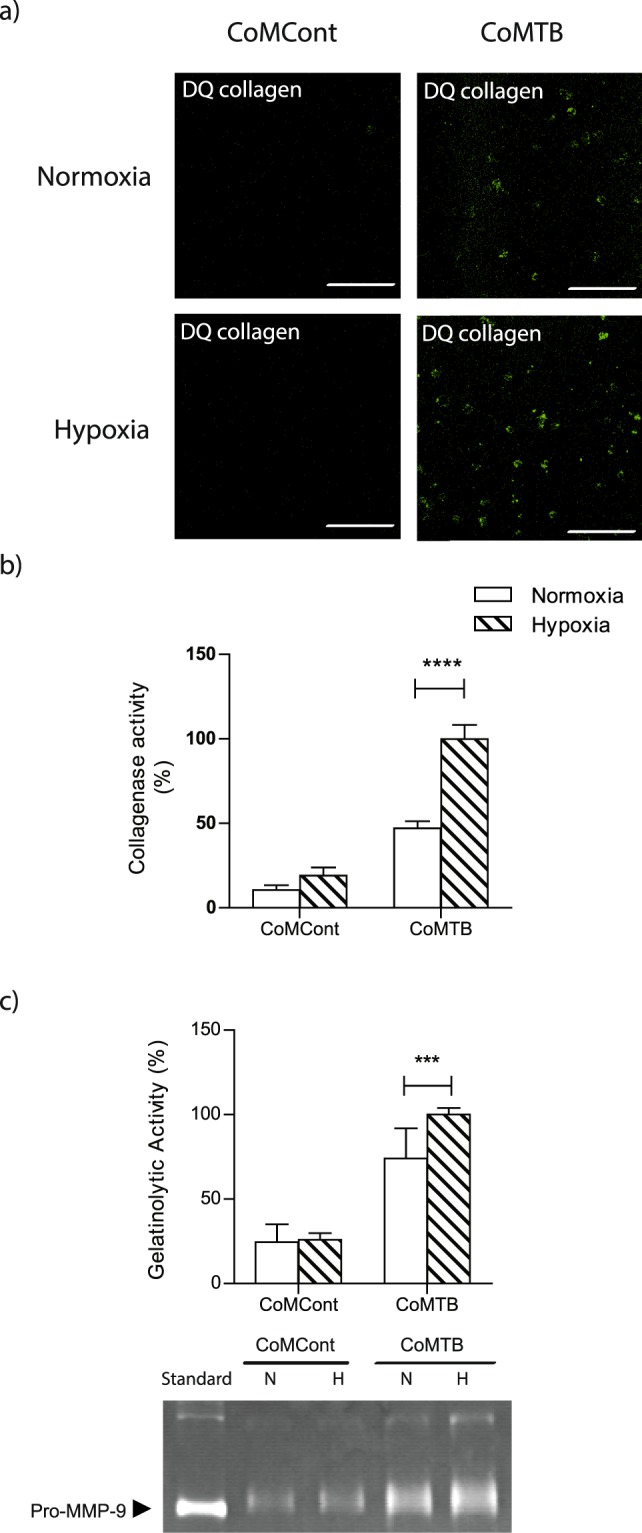
Hypoxia increases neutrophil-driven matrix destruction. (**a**) Confocal microscopy shows increased Type I collagenase activity in CoMTB-stimulated neutrophils in hypoxia compared to normoxia. (**b**) Neutrophils were stimulated with CoMCont or CoMTB and breakdown of DQ collagen analysed by quantitative fluorescent assay. (**c**) Pro-MMP-9 gelatinase activity is increased in hypoxia. MMP-9 activity by gelatin zymography with representative densitometric analysis. Zymogram representative of an experiment performed in triplicate from 2 independent experiments. Original gel is in Supplementary Figure 3. N, normoxia; H, hypoxia. ***p < 0.001, ****p < 0.0001 by 2-way ANOVA with Bonferonni post-test. Bars represent mean ± s.e.m of all data points from 3 donors each in triplicate normalized to hypoxia CoMTB.

Next, we investigated the functional effect of increased neutrophil MMP-8 secretion in hypoxia on lung matrix turnover in *M*.*tb* infection. First, using DQ Type I collagen, which fluoresces on degradation, we demonstrated that CoMTB stimulation of neutrophils increased Type I collagen degradation and this was further increased in the presence of hypoxia (Fig. 5a). This reflected an increase in collagenase activity of 112% in hypoxia (normoxia 47.1% ± 4 vs hypoxia 100% ± 8, *p* < 0.0001, Fig. 5b). CoMTB-stimulated neutrophil pro-MMP-9 gelatinase activity measured by zymography was also increased 35% in hypoxia compared to normoxia (normoxia 74.1% ± 6 vs hypoxia 100% ± 4, *p* < 0.001, Figs 5c and S3).

**Figure 6:**
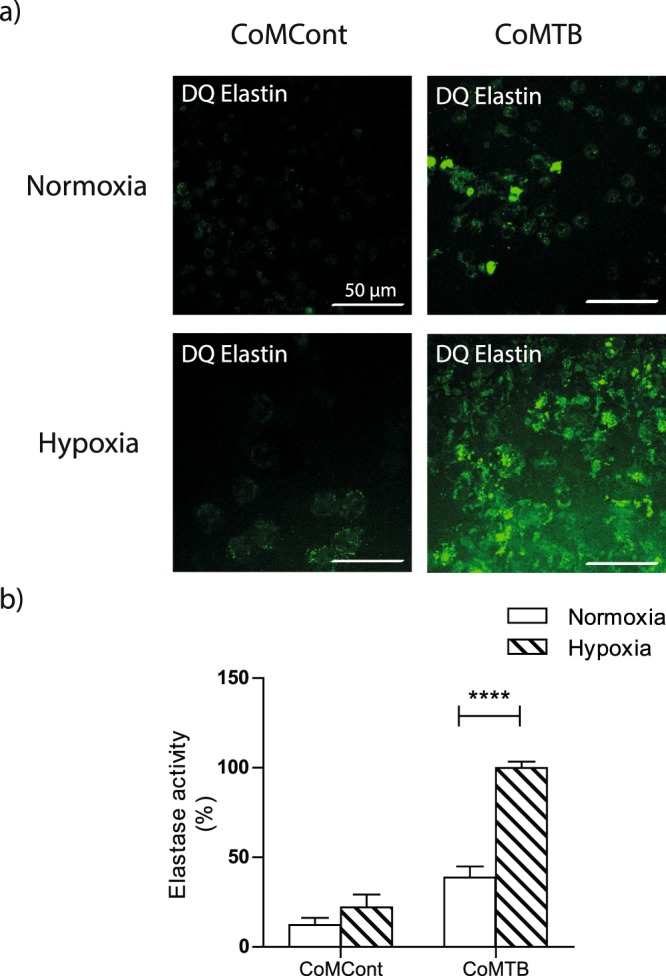
Elastin degradation is increased in hypoxia. (**a**) Neutrophils were stimulated with CoMCont or CoMTB and incubated on DQ elastin coated slides for 30 hours. (**b**) Elastase activity is increased with CoMTB in hypoxia. Breakdown of DQ elastin analysed by quantitative fluorescent assay. ***p < 0.01 by 2-way ANOVA with Bonferonni post-test. Bars represent mean ± s.e.m of all data points from 3 donors each in triplicate normalized to hypoxia CoMTB.

We further demonstrated by confocal microscopy elastin degradation in the presence of CoMTB, which was markedly increased in the presence of hypoxia (Fig. 6a). Using quantitative fluorescence assays to assess elastase activity, we confirmed an increase by 158% in degradation in hypoxia compared to normoxia (normoxia 38.7% ± 6 vs hypoxia 100% ± 3, *p* < 0.0001, Fig. 6b). Taken together, these data show that matrix destruction in TB is increased in the presence of hypoxia.

### HIF-1α stabilization increases neutrophil MMP-8 and -9 secretion

**Figure 7:**
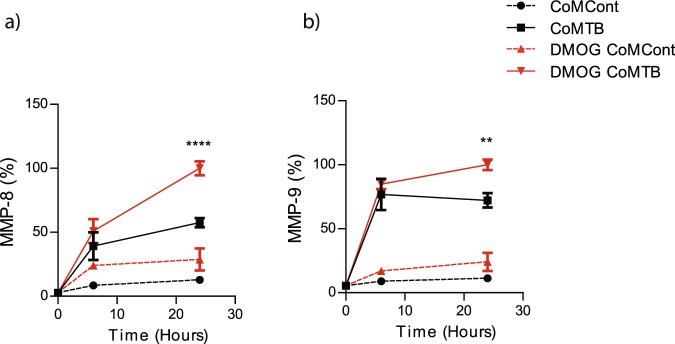
HIF-1α stabilisation modulates neutrophil MMP secretion. HIF-1α stabiliser DMOG increases (**a**) MMP-8 and (**b**) MMP-9 secretion. **p < 0.01, ****p < 0.0001 by 2-way ANOVA with Bonferonni post-test. Bars represent mean ± s.e.m of all data points from 2 donors each in triplicate normalized to DMOG CoMTB.

To investigate the mechanism by which hypoxia regulates neutrophil MMP secretion, we used dimethyloxalylglycine (DMOG) to stabilize HIF-1α expression. Neutrophils pre-treated with 1 mM DMOG prior to CoMTB stimulation secreted 74% more MMP-8 (CoMTB 57.6% ± 4 vs DMOG CoMTB 100% ± 5, p < 0.0001, Fig. 7a). DMOG similarly augmented neutrophil MMP-9 secretion by 39% with CoMTB stimulation (CoMTB 72% ± 6 vs DMOG CoMTB 100% ± 4, *p* < 0.01, Fig. 7b).

## Discussion

In this study, we investigated the impact of hypoxia on neutrophil-dependent tissue destruction in TB and show that hypoxia increases MMP-8 and neutrophil elastase secretion in the presence of CoMTB but not with *M*.*tb* stimulation. These enzymes will degrade extracellular matrix rich in types 1–3 collagen and elastin, which together are the main structural components of lung tissue. We have demonstrated that pulmonary cavities in TB patients are severely hypoxic^9^, and the consequence of hypoxia will be increased secretion of enzymes leading to more severe immunopathology. The increase in neutrophil protease secretion was not due to altered viability of CoMTB-stimulated neutrophils in hypoxia, as viability of neutrophils in hypoxia compared to normoxia was not significantly different^6,9^.

Hypoxia was demonstrated to have a functional effect with increasing MMP-8 and neutrophil elastase secretion causing destruction of extracellular matrix in TB, with an increase in gene expression of MMP-8 but not MMP-9. This is possibly due to neutrophils predominantly secreting MMP-9 pre-synthesised in granules^27^. We observed both by confocal microscopy and quantitative fluorescence assays that in hypoxia there was increased destruction of both collagen and elastin by neutrophils stimulated via monocyte-dependent networks driven by *M*.*tb* infection, although hypoxia did not alter neutrophil MMP secretion in the context of direct *M*.*tb* stimulation. This indicates that in hypoxia, neutrophils drive tissue destruction predominantly through intercellular networks. MMPs, and specifically neutrophil-derived MMPs, are emerging as key pathological mediators in diverse destructive pulmonary pathologies ranging from emphysema to TB^28–30^, with our data suggesting that hypoxia is a driver of Type I collagen, gelatin and elastin destruction, the extracellular fibrils that are key components on the human lung. However, HIF-1α also regulates antimicrobial neutrophil granule proteases production, with HIF-1α null mice demonstrating decreased neutrophil elastase, cathepsin G secretion and iNOS gene expression^31^. HIF acts as a master regulator of innates immune function in phagocytes and its expression is integral to the host defence mechanism^32^, and so the overall contribution of HIF-1α to the host-pathogen interaction in human TB will be multifactorial.

Infection with pathogens such as *Staphylococcus* and *Pseudomonas* species may increase neutrophil death, in contrast to infection with intracellular organisms such as *Anaplasma phagocytophilum* and *Leishmania* which increase neutrophil viability^33^. Although we had expected neutrophil viability to be increased by *M*.*tb*, we found that viability was not affected in normoxia. In addition, hypoxia resulted in increased neutrophil viability, independent of *M*.*tb* multiplicity of infection. Our findings are in keeping with the increase in neutrophil viability and their associated mediators in cancer, COPD and bronchiectasis, states which are associated with hypoxia and inflammation^29,34,35^.

Since the HIF-prolyl hydroxylase (PHD) pathway is a key in regulating multiple innate immune functions in hypoxia^36,37^, we investigated the role of HIF in the control of neutrophil MMP secretion in TB. We found that HIF stabilization using either physiologic hypoxia or chemical-induced HIF stabilization using DMOG increased neutrophil MMP-8. This is consistent with the finding that HIFα stabilization in a zebrafish model delays neutrophil apoptosis and increases inflammation, and our previous finding of increased MMP-1 secretion with HIF stabilization in hypoxia and with *M*.*tb* stimulation^9,20,22^. We had previously shown silencing of HIF-1α led to a decrease in MMP-1 secretion^9^, indicating that the HIF-PHD pathway regulated MMP-1 secretion, which may also regulate neutrophil MMP secretion in TB.

Pharmacologic and genetic inhibition of HIF-1α had been found to decrease NET formation^38^. We also observed that NET formation declined in hypoxia and postulate the decrease in NET formation in hypoxia may be caused by the decrease in reactive oxygen species (ROS) during hypoxia, since ROS may regulate NET formation^19,39^. The decrease in NET formation in hypoxia was not due to a decrease in neutrophil viability. The consequences of decreased NET formation in TB are uncertain although somewhat unexpectedly phagocytosis of the pathogen was not decreased.

In summary, our data demonstrate that increased neutrophil MMP-8 and elastase secretion are driven by hypoxia in our cellular model of TB. Neutrophil viability is increased in the presence of hypoxia with *M*.*tb* stimulation. The neutrophil proteases resulted in a functional increase in destruction of the lung structural matrix components collagen and elastin. Inhibiting excessive protease secretion via modulating the HIF pathway may be a potential host-directed therapeutic approach to decrease hypoxia-driven tissue destruction in TB and improve clinical outcomes particularly in the era of rising drug resistance.

## Electronic supplementary material

Supplementary Figures
