# Supplementary material for: Hypoxia increases neutrophil-driven matrix destruction after exposure to Mycobacterium tuberculosis
Source: Sci Rep. 2018 Jul 31;8:11475. doi: 10.1038/s41598-018-29659-1 (PMC6068197; doi:10.1038/s41598-018-29659-1)
Supplement: Supplementary file 1 — Supplementary Figures [file 41598_2018_29659_MOESM1_ESM.docx]

**Hypoxia increases neutrophil-driven matrix destruction after**

**exposure to *Mycobacterium tuberculosis***

**Authors:** Catherine WM Ong, Katharine Fox , Anna Ettorre, Paul T Elkington & Jon S Friedland


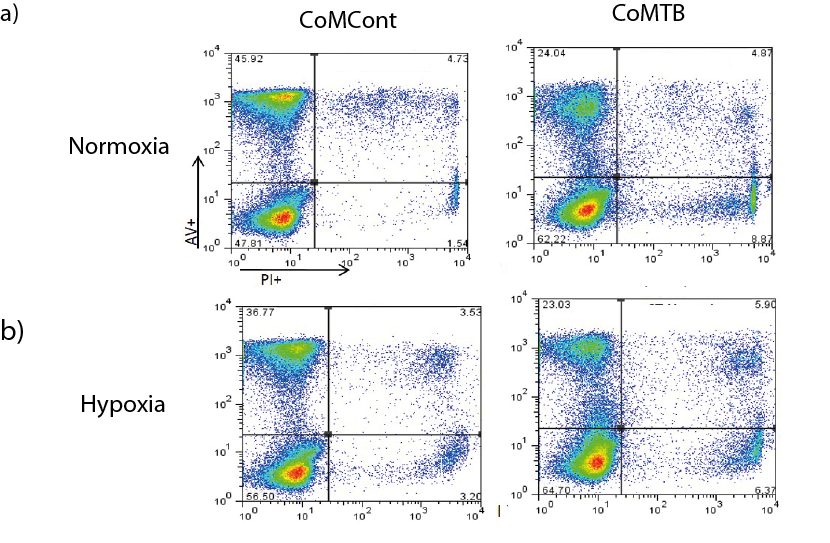


**FIGURE S1:** Hypoxia does not alter neutrophil viability with CoMTB stimulation. (a, b) Representative FACS plot of neutrophils stimulated with CoMCont or CoMTB for 30 hours and stained with Annexin V and propidium iodide. 50,000 events were collected in the gate of live cells for all FACS plots.


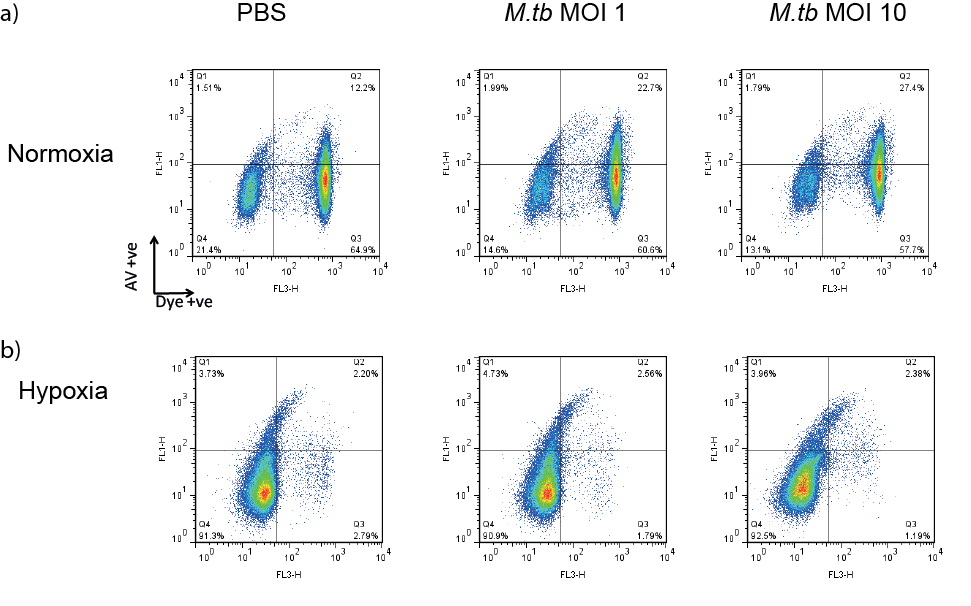


**FIGURE S2**. Hypoxia prolongs neutrophil viability in the absence and presence of *M.tb* infection. (a, b) Representative FACS plot of neutrophils stimulated with PBS, *M.tb* MOI 1 or *M.tb* MOI 10 in normoxia or hypoxia at 24 hours. In normoxia, *M.tb* infection reduced cell viability, but in hypoxia viability was increased. 50,000 events were gated. Plots representative of 4 donors.


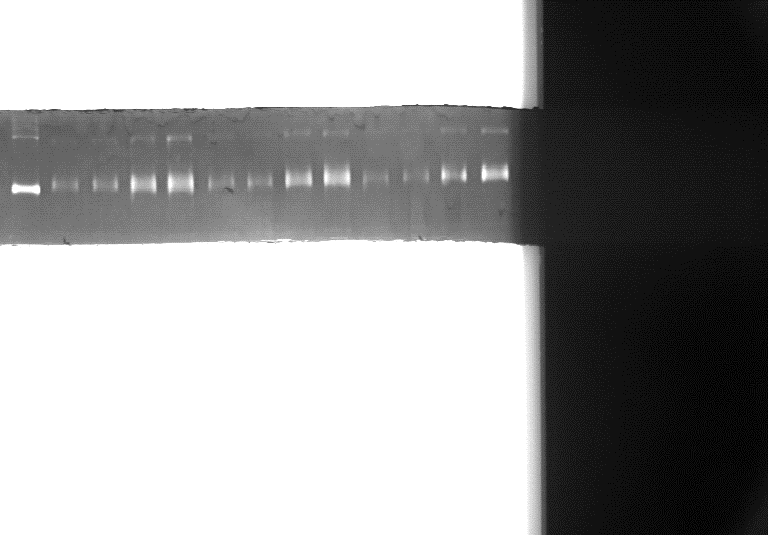


**FIGURE S3**: Uncropped version of representative gelatin zymogram shown in main figure 5c. Dashed lines correspond to areas where gel was cut.
